# Dr. Fawssett, on Spurious Vaccine

**Published:** 1801-08

**Authors:** John Fawssett

**Affiliations:** Horncastle, Lincolnshire


### *To the Editors of the Medical and Phyfical Journal.*

Gentlemen,

THREE children in this neighbourhood having lately received the Small-pox after a previous inoculation for the Vaccine difeafe, I am induced to lay the circumftances attending that that inoculation, through the medium of your Journal, before the public, to remove any doubts that may have arisen of the security of the new practice being invalidated by the accident.

On Sunday, October 19, the four children of Mr. Whitworth, of Cuxwold, in this county, were inoculated with matter received the day before from the Vaccine Institution. Owing to the struggling of one of them, (Harriet) a deeper incision was made in her arm than was wished, and it appeared the next day slightly inflamed. This inflammation was not increased on the Tuesday morning, though she complained of some pain in the arm; at night rather more inflammation was perceptible, but the part was thought to be irritated by pulling off her frock: The inflammation gradually increased on the Wednesday and Thursday, though she ceased to complain of pain from it; and on Friday a slight watery discharge took place from the inoculated part. This continued, as did the extension of the inflammation, during Saturday and Sunday. On Sunday morning she took some doses of an aperient mixture, which operated mildly, and in the afternoon she appeared to be slightly feverish. On Monday the discharge from the arm was diminished, and the inflamed areola, which was scarcely of the diameter of half a crown, seemed at a stand. On Tuesday a scab had formed, covering a little purulent matter; from this time the inflammation subsided, and a dry dark coloured scab, such as commonly succeeds to a Cow-pox pustule, continued on the arm for some weeks.

No distinct vesicle or pustule was ever formed.

The eldest and youngest child were not at all affected by the inoculation.

The arm of the remaining child (Georgiana) was thought to be slightly inflamed on the day succeeding inoculation, but this was not perceptible on either Tuesday or Wednesday. Early on Thursday morning she rubbed it a good deal; at noon it was inflamed, and a minute vesicle was visible on the inoculated part: The inflammation gradually extended itself, and on the Saturday there was a watery discharge from the vesicle. On Monday the discharge was lessened, and the inflammation, which was about the breadth of a shilling, seemed at a stand. From that time both gradually abated, and were succeeded by a scab exactly similar to that on Harriet's arm.

On Thursday, October 30, Mary and William were again inoculated with matter taken on a thread, at the beginning of the second week, from Harriet's arm. The plaster was removed on the third day, and no inflammation was visible on the arm of Mary, though a slight one appeared on that of William. The inflammation extended itself on the arm of the the latter in the ſame gradual manner it had done on thoſe of his ſiſters, and on the ſixth or ſeventh day a watery diſcharge took place. He was never feveriſh during the progreſs of it, though he had been much out of health the week or two before; and about the ninth day the inflammation, which was rather larger than a ſhilling, became ſtationary; the diſcharge gradually ſubſided, and the ſcab formed preciſely as on the other two. No puſtule or veſicle was ever formed in this caſe.

The inoculation of Georgiana, in whom the inflammation had apparently been excited by her rubbing her arm, was again repeated, and of Mary a third time, with matter taken from their brother, but entirely without effect.

It was inconvenient to the family, who were brought to this town for the purpoſe of inoculation, to remain longer here; and all but Mary, on whom every attempt to produce infection had been fruitleſs, were removed.

The point in which the caſes above detailed principally deviated from the regular appearance of the Vaccine diſeaſe, was in the abſence of the very characteriſtic veſicle* or puſtule, ſo well known to every one who has had an opportunity of witneſſing the complaint, and ſo admirably delineated in Dr. Jenner's publication.

The inflamed areola too was rather ſmall in each of them, and became ſtationary ſomewhat earlier I think than it generally does. I could not diſcern any variety of tinge in the inflammation, as ſome of your correſpondents have noticed in irregular caſes, nor did I obſerve that the hardneſs round the inoculated part was later in its appearance than uſual. The matter diſcharged from the ſore was at firſt perfectly limpid.

The early appearance of inflammation might, perhaps, be objected to, in the caſe of Harriet; but it was ſo very ſlight, and did not increaſe till the third day, that it was attributed to the puncture being accidentally deeper than was intended, and it was not expected that the nature of the diſeaſe could be altered by that circumſtance.

The inflammation not appearing on Georgiana's arm till ſhe had rubbed it a good deal, on the fourth day, rendered the reſult of her inoculation particularly unſatisfactory, and it was accordingly repeated, but without effect. The after progreſs of

* Georgiana, it was mentioned, had a minute veſicle on the inoculated part of her arm; but it was more tranſparent, more globular, and appeared to have a thinner pellicle than marks the moſt decided Cow-pox puſtule.

of the inflammation on her arm was gradual, and precifely as the reft.

The abfence of the veficle appeared to me the moft important deviation from the ordinary progrefs of the difeafe, and enough to render the efficacy of the inoculation, in all of them, ambiguous; but as two cafes occurred here two years ago, in which it was equally wanting, and the patients were neverthelefs unaffected by a fubfequent Small-pox inoculation,* I was in hopes our prefent patients would have enjoyed the fame fecurity. At the fame time, could any recent matter have been then obtained, I fhould certainly have wifhed to repeat the inoculation in every one of them. That opportunity, however, was not afforded me; and a general inoculation for Small-pox taking place foon after in the village where they lived, they, with the reft, were fubjected to it, and received the difeafe in a very mild and regular manner.

The failure of the above cafes, though from the circumftances here mentioned it was, in two of them at leaft, unexpected, ftill cannot be confidered by any one who has paid due attention to the fubject, as invalidating the fecurity of the Cow-pox inoculation, where the progrefs of it has been unequivocal. It teaches us, indeed, that a flight deviation from the regular appearances may affect that fecurity, and where a favourable repetition of inoculation is not in our power, muft of courfe, induce us to with-hold our refponfibility for its efficacy in fuch inftances. It ftrongly enforces too the neceffity of minute attention to the progrefs of the local affection, as the only criterion of a fuccefsful reception of it; fever, tendernefs in the axilla, and other more certain fymptoms of conftitutional affection fo frequently being wanting; nor, as appears from one of the above cafes, does a flight appearance of the former afford any additional fecurity, where the local affection is not fatisfactory.

In the country, we labour under great inconveniences in feldom being able to obtain recent matter, that fent from a diftance fo often failing to produce the difeafe, that little reliance can be placed on it. Several times it has lately happened in this neighbourhood, that the matter received from the Vaccine Inftitution has either failed to produce any inflammation, or, as in thefe cafes, only occafioned an imperfect form of the difeafe.

* The patients above alluded to were inoculated by a thread, and as the plafter was not removed before the fourth day, the veficle might be broken in doing it. In feveral other cafes, though managed in the fame way, that did not happen.

difeafe. Till better means therefore of preferving the matter are difcovered, the progrefs of the Vaccine Inoculation in the country will, I fear, be but flow, notwithftanding every day's experience fhould continue to confirm its importance.

The eldeft of the children who was left at Horncaftle, was inoculated fome weeks afterwards with recent matter procured in the neighbourhood, had the difeafe in its moft decided form, and has fince been twice inoculated for Small-pox without any effect.

One of your correfpondents has remarked, that perfons with a fmooth clear fkin, flaxen hair, and light eyes, are leaft fufceptible of Vaccine infection; thefe children were precifely of this temperament.

*Horncastle, Lincolnshire,* I am, &c.

*July* 7, 1801. JOHN FAWSSETT.

The Editors perceive that Dr. Fawffett's ideas on this fubject are perfectly correct, and affure him that *proper* virus, inclofed between two glafs plates, may be fent to China, and never fail to communicate the difeafe. This method of fending virus to a diftance is always employed by Dr. Jenner, who never ufes any that is not taken before the areola is fully formed. When taken later it is not to be depended upon as a preventive of Small-pox, although it will produce a difeafe imitative of the true Vaccine.

